# Editorial: Farm animal welfare and climate change: methods of assessment and mitigation

**DOI:** 10.3389/fvets.2023.1343934

**Published:** 2024-01-05

**Authors:** Ikram Ben Souf, Mariem Saidani, Manel Ben Larbi, Naceur M'Hamdi

**Affiliations:** ^1^Research Laboratory of Ecosystems and Aquatic Resources, UR03AGRO, National Agronomic Institute of Tunisia, University of Carthage, Tunis, Tunisia; ^2^Research Unit of Biodiversity and Resource Development in Mountain Areas of Tunisia, UR17AGR14, Higher School of Agriculture of Mateur, Carthage University, Tunis, Tunisia

**Keywords:** animal welfare, stress, mitigation, indicators, measurement

## Introduction

In recent years, the intersection of farm animal welfare and climate change has emerged as a critical area of concern in the field of veterinary sciences (1, 2). As the global population continues to grow, the demand for animal products escalates, placing unprecedented pressure on both livestock and the environment (3, 4). The welfare of farm animals is not only an ethical imperative but also a key determinant of their productivity and environmental impact (5). Climate change adds an additional layer of complexity, as rising temperatures, changing precipitation patterns, and extreme weather events pose new challenges to animal husbandry practices (6, 7). This Research Topic delves into the multifaceted aspects of this nexus, addressing the urgent need for innovative solutions to safeguard both animal wellbeing and the planet.

The Research Topic of Frontiers in Veterinary Sciences aims to explore the intricate relationship between farm animal welfare and climate change, focusing on cutting-edge methods of assessment and mitigation strategies.

The Research Topic of Frontiers in Veterinary Sciences, titled “*Farm animal welfare and climate change: methods of assessment and mitigation*,” has five groundbreaking pieces that address the vital junction of farm animal welfare and climate change. The issue delves into novel approaches to assessing and mitigating the effects of climate change on livestock, emphasizing the significance of sustainable agricultural practices. These papers provide vital insights into the developing landscape of veterinary sciences by investigating creative techniques to protect both farm animal welfare and the environment. The interdisciplinary nature of the research presented emphasizes the importance of implementing comprehensive strategies to address the complex challenges arising from the intersection of farm animal welfare and climate change, fostering a comprehensive understanding that can inform future policy and practice in the field.

In the first paper of Cheng et al. “*High genetic merit dairy heifers grazing low quality forage had similar weight gain and urinary nitrogen excretion to those of low genetic merit heifers*,” researchers investigated the intriguing relationship between genetic merit, forage quality, and physiological outcomes in dairy heifers. This research looks into the effect of genetic potential on weight increase and urinary nitrogen excretion, both of which are important elements in bovine growth and environmental sustainability. The findings defy conventional wisdom by demonstrating that despite feeding on low-quality fodder, heifers with high genetic merit displayed equivalent weight gain and urinary nitrogen excretion to their low-genetic-merit counterparts. This suggests that genetic factors may play a nuanced role in nutrient utilization and metabolic efficiency, providing valuable insights for optimizing dairy industry livestock management practices and resource utilization.

The paper by Bakony et al. “*The use of body surface temperatures in assessing thermal status of hutch-reared dairy calves in shaded and unshaded conditions*” looks into the effect of environmental conditions on the thermal wellbeing of hutch-reared dairy calves. The study focuses on the use of body surface temperatures as a critical metric for assessing the thermal status of calves. The research aims to determine the impact of temperature regulation on the health and welfare of calves by conducting experiments in both shaded and unshaded environments. The findings could have implications for dairy farming practices, such as optimizing conditions for hutch-reared calves and improving overall animal welfare. This study adds valuable knowledge to the intersection of animal science and husbandry practices, addressing an important aspect of the agricultural industry's commitment to promoting livestock wellbeing.

The original study of Escribano et al., on the changes in cortisol and cortisol levels in the hair of heat-stressed pigs, investigates the physiological response of pigs to elevated temperatures. The researchers want to know how heat stress affects the concentrations of cortisol and cortisol, stress and metabolic regulation hormones, in pig hair. Researchers investigate the potential biomarkers in hair as indicators of chronic stress caused by prolonged exposure to high temperatures using controlled experiments and meticulous sample analysis. This study not only helps pigs in agricultural settings, but it also has broader implications for understanding stress-related hormonal changes in animals. The findings could help researchers develop effective strategies for mitigating heat stress and improving livestock wellbeing and productivity in the face of climate-related challenges.

The paper of Fu et al., on the *Effects of short-distance transportation on physiological indexes, intestinal morphology, microbial community, and the transcriptome of the jejunum in weaned piglets* represents a comprehensive investigation into the multifaceted effects of transportation stress on these animals' health and wellbeing. The research looks at physiological markers to assess stress responses, changes in intestinal morphology to understand potential changes in digestive function, changes in microbial communities to assess gut health, and the transcriptome of the jejunum to uncover molecular mechanisms underlying stress-related responses. The research aims to provide a nuanced understanding of how short-distance transportation can influence various aspects of piglet physiology, providing valuable insights for optimizing transport conditions and enhancing the overall welfare of weaned piglets in the swine industry by examining these diverse parameters.

The review paper of Shah et al., provides a thorough examination of this remarkable animal's life in harsh environments, including a comprehensive overview of its feeding habits, growth patterns, production performance, and significant contributions to food security. The paper sheds light on the unique adaptations that allow yaks to thrive in challenging climates by synthesizing existing research, and emphasizing their role as resilient contributors to sustainable agriculture. The report delves into the complexities of yak feeding habits, describing their ability to graze on diverse, often sparse vegetation. Furthermore, it investigates yak growth trajectories and their critical role in local economies by elucidating their production performance, which includes meat, milk, and fiber yield. Finally, the review emphasizes the importance of yaks in ensuring food security, especially in areas where conventional livestock may struggle, and provides valuable insights for researchers, policymakers, and practitioners in the field.

## Conclusion

The purpose of this Research Topic is to promote a thorough understanding of the dynamic interplay between farm animal welfare and climate change. It aims to provide valuable insights for veterinarians, researchers, policymakers, and industry stakeholders committed to promoting a more sustainable, humane, and resilient future for livestock farming by featuring the most recent research and innovations. The veterinary community can make a significant contribution to mitigating the impact of climate change on farm animals while ensuring their wellbeing in an evolving global landscape through collaborative efforts, informed research, and a commitment to responsible practices.

## Author contributions

IS: Writing – original draft, Methodology. MS: Conceptualization, Writing – original draft. MB: Investigation, Writing – review & editing. NM'H: Investigation, Supervision, Validation, Visualization, Writing – review & editing.
